# Api5 Contributes to E2F1 Control of the G1/S Cell Cycle Phase Transition

**DOI:** 10.1371/journal.pone.0071443

**Published:** 2013-08-07

**Authors:** Marina Garcia-Jove Navarro, Céline Basset, Tania Arcondéguy, Christian Touriol, Guillaume Perez, Hervé Prats, Eric Lacazette

**Affiliations:** INSERM UMR 1037, Cancer Research Center of Toulouse (CRCT), Cancer Department, Toulouse, France; University of Newcastle upon Tyne, United Kingdom

## Abstract

**Background:**

The E2f transcription factor family has a pivotal role in controlling the cell fate in general, and in particular cancer development, by regulating the expression of several genes required for S phase entry and progression through the cell cycle. It has become clear that the transcriptional activation of at least one member of the family, E2F1, can also induce apoptosis. An appropriate balance of positive and negative regulators appears to be necessary to modulate E2F1 transcriptional activity, and thus cell fate.

**Methodology/Principal Findings:**

In this report, we show that Api5, already known as a regulator of E2F1 induced-apoptosis, is required for the E2F1 transcriptional activation of G1/S transition genes, and consequently, for cell cycle progression and cell proliferation. Api5 appears to be a cell cycle regulated protein. Removal of Api5 reduces cyclin E, cyclin A, cyclin D1 and Cdk2 levels, causing G1 cell cycle arrest and cell cycle delay. Luciferase assays established that Api5 directly regulates the expression of several G1/S genes under E2F1 control. Using protein/protein and protein/DNA immunoprecipitation studies, we demonstrate that Api5, even if not physically interacting with E2F1, contributes positively to E2F1 transcriptional activity by increasing E2F1 binding to its target promoters, through an indirect mechanism.

**Conclusion/Significance:**

The results described here support the pivotal role of cell cycle related proteins, that like E2F1, may act as tumor suppressors or as proto-oncogenes during cancer development, depending on the behavior of their positive and negative regulators. According to our findings, Api5 contributes to E2F1 transcriptional activation of cell cycle-associated genes by facilitating E2F1 recruitment onto its target promoters and thus E2F1 target gene transcription.

## Introduction

An equilibrated balance between cell proliferation and apoptosis is required for organism development and homeostasis. A deregulation between these two critical processes can lead to multiple pathologies, the most frequent being cancer [1] [2]. The E2-promoter binding factor (E2F) family participates in the control of this balance as its members regulate both processes, depending on the biological context [3] [4]. The E2F family consists of 8 members traditionally divided into activator (E2F1, E2F2, E2F3a and E2F3b) and inhibitor (E2F4, E2F5, E2F6, E2F7a/b) subclasses [5]. Most E2Fs form active heterodimers with a member of the DP protein family, namely DP1 or DP2. However, the transcriptional activity of the complex is carried out by the E2F protein [4].

The first member of the family to be discovered, E2F1, is a critical target of the retinoblastoma tumor suppressor protein (pRb) [6] [7] [8]. The best documented activity of E2F1 is the transcriptional regulation of a dozen genes involved in cell cycle progression [9]. Mitogenic signals trigger E2F1 activation leading to the transcription of genes encoding proteins required for G1/S phase transition and DNA synthesis, such as cyclin E, cyclin A, Cdk2, cdc25 or SKP2 [10] [11] [12] [13]. In a non-proliferating context, pRb interacts with DNA-bound E2F1, preventing its transcriptional activity necessary for the G1/S phase transition [14]. As the pRb pathway is functionally inactive in most tumor cells, this can result in deregulation of E2F1 activity, leading to uncontrolled cell proliferation [15].

On the contrary, much data from the literature indicate a role for E2F1 during programmed cell death [16] [17]. Ectopic expression of E2F1 induces S-phase entry and subsequently leads to apoptosis [18] [19] [20]. In addition, E2F1 deficient mice suffer a lack of apoptosis and aberrant cell proliferation [21]. To date, the balance between cell survival and cell death controlled by E2F1 is still poorly understood and needs further investigation.

In this context, a study by Morris *et al*., identified a protein named Apoptosis inhibitor-5/antiapoptotic clone-11/Fibroblast Growth Factor-2 Interacting Factor (Api5/aac11/FIF) as a critical determinant of E2F1 induced apoptosis *in vivo* and *in vitro* [22]. While there is a plethora of information in the literature on E2F1, very little is known about Api5 function.

Api5 is a nuclear protein initially identified for its anti-apoptotic function. Api5 overexpression prevents apoptosis after serum and growth factor starvation [23]. Recently, Api5 has been shown to bind Acinus, which prevents Acinus-mediated DNA fragmentation, and thus, apoptosis [24]. Van den Berghe *et al*, 2000 identified Api5 as a binding partner of the prosurvival growth factor FGF-2 [25]. Interestingly, Api5 expression is deregulated in multiple cancer cell lines, including various human cancers: cervical or prostate, Non Small Cell Lung Cancer (NSCLC), and B cell chronic lymphoid leukemia (B-CLL) [23] [26] [25] [27] [28] [22] [29] [24] [30]. In NSCLC, Api5 overexpression has been associated with poor survival of patients [26]. Moreover, Api5 depletion has been shown to be lethal to tumor cells under low serum stress [27], whereas Api5 overexpression promotes cell growth and migration. It has also been reported that the lack of miR-143 and miR-145 expression induces Api5 up-regulation in ulcerative colitis, a disease predisposing to colon cancer [31]. The literature so far strongly suggests that Api5 plays a pivotal role in cell fate. These data suggest that Api5 could be considered as a putative oncogene.

Although the link between Api5 and E2F1 in the apoptosis pathway has been elegantly described [22], it is not known whether Api5 and E2F1 could have a dual function and participate in a common cell cycle regulation pathway. To address this question, we studied Api5 expression in synchronized cells and found that Api5 is differentially expressed during the cell cycle. Furthermore, Api5 is required for normal cell cycle progression and cell proliferation. Our data indicate that Api5 contributes functionally to cell progression through G1 into the S phase, by altering G1/S transition gene expression of E2F1 regulated genes. We showed by chromatin immunoprecipitation experiments that Api5 increases E2F1 recruitment onto its target promoters.

## Results

### Api5 Knockdown Leads to Cell Cycle Arrest in G1 Phase

**Figure 1:**
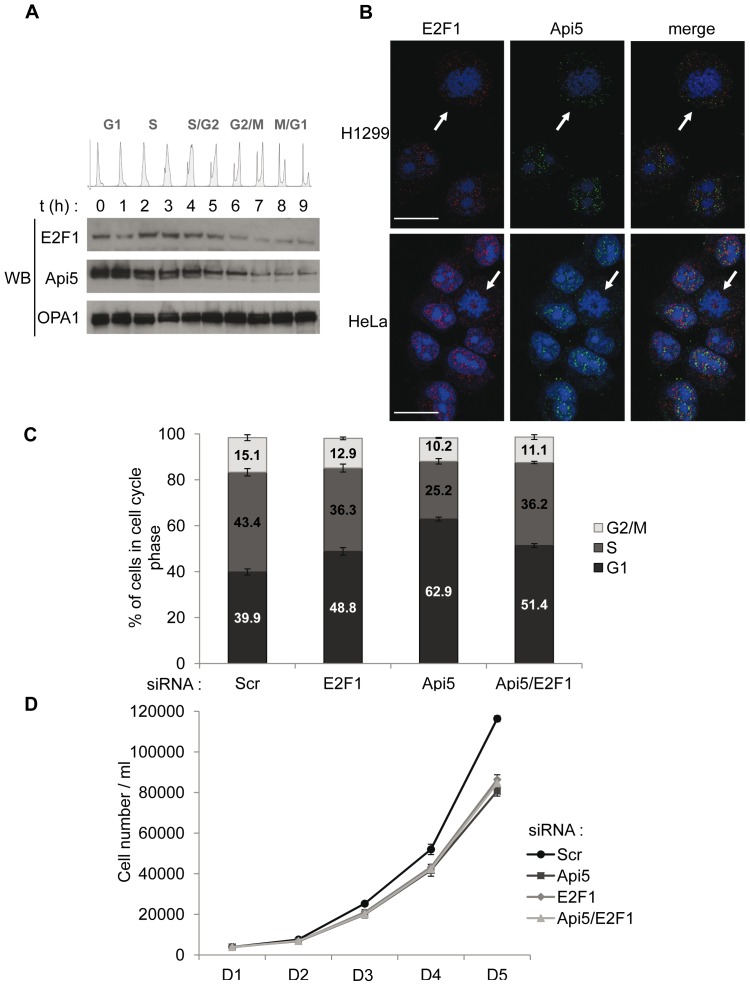
Api5 depletion induces cell accumulation in G1 phase and decreases cell proliferation. **A.** H1299 cells were synchronized by a double thymidine block, released and collected (Figure S1). Api5, E2F1 and OPA1 expression through the cell cycle was analyzed by Western blot (WB). **B.** Endogenous Api5 (green) and E2F1 (red) immunostaining were performed on H1299 and HeLa cells; nuclei were stained with PI (blue). White arrows show cells undergoing mitosis. [Scale bar 20 µm]. **C.** Cytometric quantification of experiments described in (A). Cell cycle analysis was performed using FlowJo. **D.** Cell growth was analyzed by cell counting. H1299 cells were transfected by the indicated siRNAs.

Expression of most cell cycle regulators, including E2F1, is modulated throughout the cell cycle in order to regulate their activity. To better define the relationship between cell cycle related E2F1 function and Api5, we investigated whether Api5 protein levels were also cell cycle regulated. H1299 cells were synchronized in G1 phase by a double-thymidine block (Figure 1A, 0 h). They were then released into growth media containing 10% FBS to stimulate the cells to re-enter the cell cycle (Figure 1A, 1 h–9 h). Every hour, cells were harvested and prepared either for cell cycle analysis (Figure S1) or Western blot analysis (Figure 1A) in order to compare Api5 and E2F1 expression patterns throughout the cell cycle phases. Western blot analysis revealed that Api5 and E2F1 were both periodically expressed throughout the cell cycle. The E2F1 pattern was in accordance with the literature [32] [33]. Remarkably, Api5 exhibited an expression profile over time that was partially coordinated to that of E2F1. The level of Api5 protein peaked at the end of the G1 phase and was significantly reduced, but stabilized, throughout the S phase, while the E2F1 protein level also peaked approaching the G1/S phase transition, but remained high during the S phase (Figure 1A, t = 0 h–4 h). Both Api5 and E2F1 protein levels then started to decrease in the G2 phase and become minimal during the M phase (Figure 1A, t5 h–t9 h). OPA1, a mitochondrial protein, was used as loading control. The Western blot results were confirmed by immuno-fluorescent staining in H1299 and HeLa cells: Api5 and E2F1 were almost undetectable when the cells underwent mitosis (Figure 1B, white arrows). Moreover, we observed that Api5 and E2F1 did not co-localize in the nuclei of HeLa and H1299 cells (Figure 1B, merge).

The concomitant expression of Api5 and E2F1 during the cell cycle and their functional relationship described by Morris *et al.* in a previous study [24] suggested that Api5 could have a cell cycle related function. To test this hypothesis, we used flow cytometry to determine whether Api5 inhibition may impair cell cycle progression. For this purpose, H1299 cells were transfected with Api5, E2F1, Api5/E2F1, or scrambled siRNAs. The cell cycle phase distribution was then analyzed (Figure 1C and Figure S2). As expected, E2F1 knockdown led to a significant increase (8.9%) in cells in the G1 phase compared to the control experiment. This increase was most likely due to the lack of E2F1 transcriptional induction of its G1/S transition target genes. As a consequence, the proportion of cells in S phase and in G2/M phases decreased by 7.1% and 2.2%, respectively. Interestingly, Api5 knockdown also induced G1 accumulation that was much higher than the effect induced by E2F1 depletion (23% versus 8.9%). Consequently, the percentage of cells in S phase was drastically reduced from 43.4% to 29.4%, when compared to the control condition, as was the percentage of cells in G2/M phases (from 15.1% to 10.2%). The effect of Api5 and E2F1 double depletion on cell cycle phase distribution was also analyzed. As shown in figure 1C, no cumulative effect was observed. The proportion of treated cells in G1 phase increased by 11.5%, leading to a decrease in cells in S and G2/M phases of 7.2% and 4% respectively compared to control cells.

Furthermore, to assess if the G1 arrest observed after Api5 depletion affected cell proliferation, H1299 cell growth was analyzed after Api5, E2F1, Api5/E2F1, or scrambled siRNAs transfection. As expected, knockdown of E2F1 or Api5 markedly inhibited cell proliferation (Figure 1D), though the double E2F1 and Api5 knockdown did not amplify the effect observed with single siRNA treatments.

These results revealed that Api5 is necessary for a normal cell cycle progression at the G1/S phase transition and cell proliferation. As no cumulative effect was observed when using both Api5 and E2F1 siRNAs, Api5 could positively control the cell cycle and cell proliferation through the E2F1 regulatory pathway.

### Api5 Contributes to the Expression of E2F1-target Genes Involved in G1/S Transition

**Figure 2:**
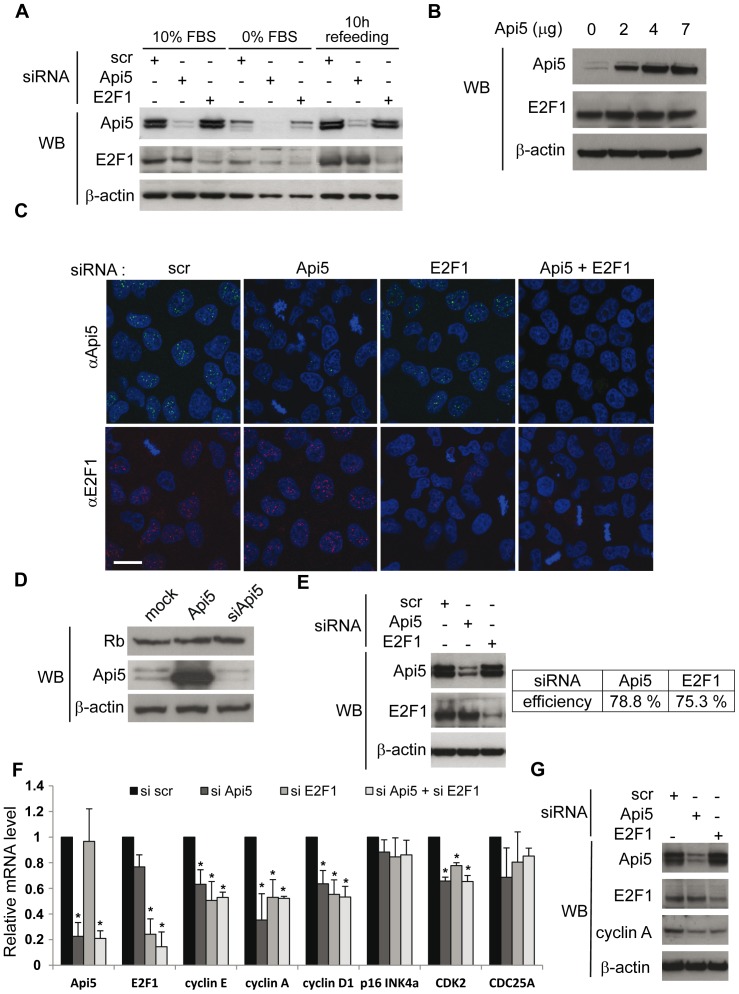
Api5 depletion induces a decrease in E2F1-target gene mRNA levels without affecting E2F1 expression. **A.** Api5 and E2F1 reciprocal regulation. After depletion of Api5 and E2F1 with specific siRNAs, H1299 cells were made quiescent by serum starvation for 48 h (0% FBS) and then allowed to progress through the cell cycle over 10 hours by FBS addition (10 h refeeding). Control cells were grown in 10% fetal bovine serum media (10% FBS). Api5, E2F1 and β-actin expression was analyzed by Western blot (WB). **B.** E2F1 and β-actin expression was analyzed by Western blot (WB) after Api5 overexpression. HeLa cells were transfected with different amounts of a plasmid encoding Api5. **C.** Api5 and E2F1 immunocytochemistry. HeLa cells were transfected with the indicated siRNAs or treated with etoposide for 16 hours. Endogenous immunodetection of Api5 (green) and E2F1 (red). Nuclei were stained with PI (blue). [Scale bar 20 µm]. **D.** Retinoblastoma expression under Api5 overexpression or depletion. Hela cells were transfected or not with a plasmid encoding Api5 or an siRNA directed against Api5. Western blot analysis was performed with an anti-retinoblastoma antibody. **E.** siRNA efficiency of quantitative PCR samples. HeLa cells were treated with Api5 or E2F1 siRNAs. Api5 and E2F1 protein content was assessed by Western blot (left panel) to evaluate siRNA efficiencies by densitometric analysis (relative to scrambled treated cells) (right panel). **F.** E2F1 target gene expression. HeLa cells were treated with Api5 or E2F1 siRNA, or with both siRNAs. Api5, E2F1, cyclin E, cyclin A, cyclin D1, p16 INK4a, Cdk2 and Cdc25A relative mRNA levels were measured by quantitative PCR, using HPRT1 and GUSB mRNA levels as references. Asterisks indicate a significant decrease compared to control conditions (scrambled siRNA treated cells). Statistical significance was determined by two-tailed Student’s *t* test (*, p<0.05). **G.** Cyclin A expression. Western blot analysis of cellular levels of cyclin A protein after transfection of H1299 cells with Api5 or E2F1 siRNAs.

Our results showed that the Api5 protein level increased as cells progressed through G1 phase to G1/S transition while Api5 depletion drastically impaired cell cycle progression and cell proliferation. To understand Api5 function during G1/S phase transition, we first examined if Api5 could affect the E2F1 protein G1/S accumulation needed for G1/S cell cycle phase transition. The absence of data concerning a possible regulation of Api5 expression mediated by E2F1, or the opposite, led us consider these hypotheses. H1299 cells were made quiescent, referred to as cells in G0-like phase, by serum starvation. Api5 and E2F1 protein levels were lower in quiescent cells (Figure 2A, 0% FBS) when compared to normal cycling cells (Figure 2A, 10% FBS). As expected, when the cells were allowed, by serum refeeding, to progress through G1 phase and to pass through the restriction point into G1/S transition (Figure 2A, 10 h after refeeding), Api5 and E2F1 reached their original protein levels. In fact, Api5 and E2F1 expression was detectable in mid-G1 of cycling cells after 7 hours of serum refeeding (data not shown) and the protein levels increased as cells approached the G1/S phase transition (Figure 2A, 10 h after refeeding). To shed light on a possible reciprocal regulation of Api5 and E2F1, induction of the G1/S phase transition was examined in cells transiently transfected with Api5 or E2F1 siRNAs (Figure 2A). Western blot analysis showed that neither E2F1 knockdown affected Api5 protein induction nor did Api5 knockdown affect E2F1 induction approaching G1/S transition. These results showed that Api5 and E2F1 were not involved with each other in the up-regulation approaching G1/S phase transition.

Additionally, to determine whether E2F1 expression could be modified by an abnormal increase of Api5, HeLa cells were transiently transfected with different amounts of expression vector encoding Api5 (Figure 2B). No variation in the E2F1 protein level was observed by Western blot analysis (Figure 2B). Thus, E2F1 is neither up-regulated, nor altered in response to Api5 overexpression.

Taken together, these results indicate that Api5 and E2F1 are not involved in any reciprocal control at the expression level.

Western blot analysis revealed the two previously characterized splicing isoforms of Api5, namely the 504 and 510 amino acid isoforms [25]. Both isoforms decreased when cells were transfected with the specific Api5 siRNA (targeting both isoforms) and evolved similarly during cell cycle (Figures 1A and 2A). In addition, we detected only one specific band of endogenous E2F1, which decreased when cells were transfected with the specific E2F1 siRNA.

As Api5 and E2F1 clearly do not affect their reciprocal expression, we considered the possibility that Api5 could affect E2F1 nucleocytoplasmic shuttling and thus its nuclear accumulation. However, Api5 depleted HeLa cells were not affected by E2F1 nulear localization (Figure 2C). Reciprocally, E2F1 depleted cells did not affect Api5 nuclear levels (Figure 2C).

In addition, the level of pRb protein, the main E2F1 activity regulator, was not affected by Api5 overexpression or Api5 knockdown (Figure 2D).

The progression of cells through G1 and into S phase coincides with the temporal expression of genes whose products are required for the next phase of the cell cycle [32]. It is well established that E2F1 directly trans-activates numerous target promoters, resulting in the synthesis of proteins required during cell cycle progression (including cyclin D1, Myc, cyclin E, SKP2, Cdk2, cyclin A), DNA synthesis and replication (e.g. MCM2-7, CDC6, TK), and checkpoints (e.g. BRCA1-2, TP53) [32] [34] [35] [36] (for review refer to [4]). In order to determine if Api5 may contribute to transcriptional activity of E2F1, HeLa cells were transiently transfected with Api5 or E2F1 siRNAs (Figure 2E) and the mRNA level of different E2F1-target cell cycle regulator genes was analyzed by quantitative RT-PCR (Figure 2F). Interestingly, Api5 knockdown induced a significant down-regulation of cyclin E, cyclin A, cyclin D1 and Cdk2 mRNA levels, but not of p16 INK4a and CDC25A mRNA. Similar results were obtained with the E2F1 knockdown (Figure 2F). Consistent with the Western blot results shown in Figure 2A, the decrease observed for cyclin E, cyclin A, cyclin D1 or Cdk2 mRNA levels when Api5 was depleted, was unrelated to a significant decrease in E2F1 mRNA (Figure 2F). Additionally, no additive effect was observed when both proteins (Api5 and E2F1) were depleted (Figure 2F). These findings reinforce the hypothesis that Api5 and E2F1 belong to the same molecular pathway.

In addition, we analyzed the consequences of the Api5 or E2F1 down-regulation on Cyclin A protein levels by Western blot analysis, as this target had the strongest response in quantitative PCR experiments. As expected, Api5 or E2F1 suppression similarly lowered the level of cyclin A protein by about 50% (Figure 2G).

Taken together, these results indicate that Api5 enhances the expression of E2F1-target genes involved in the G1/S transition without affecting E2F1 mRNA and protein levels.

### Api5 Modulates the Transcriptional Activation by E2F1 of Distinct G1/S Phase Transition Genes

To investigate if Api5 could act as a transcriptional modulator of E2F1 target genes involved in G1/S phase transition genes, we carried out luciferase reporter assays under the control of E2F1 responsive promoters.

**Figure 3:**
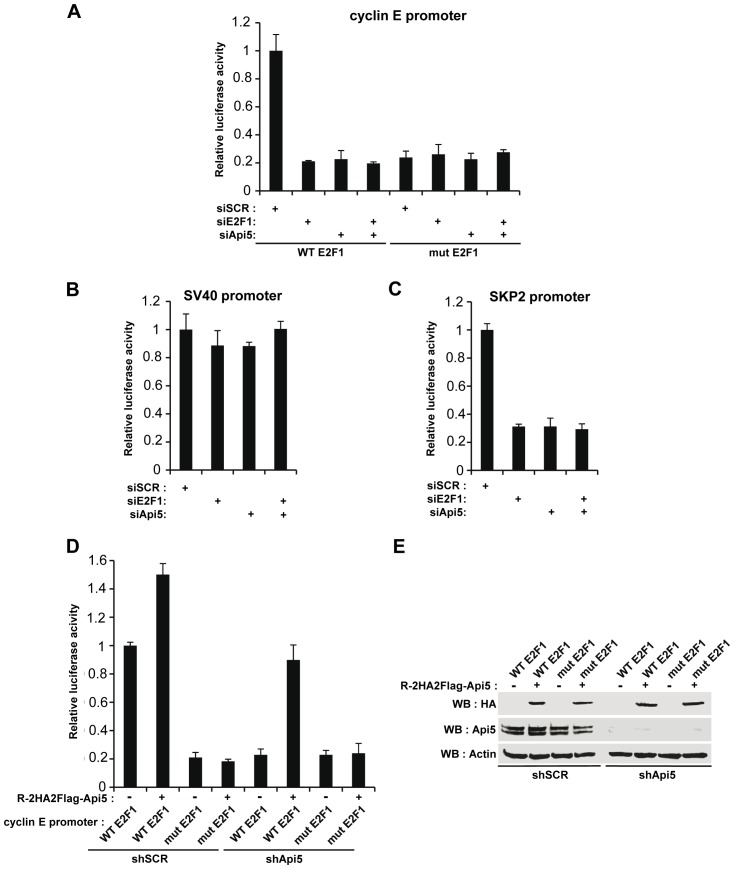
Api5 stimulates the transcriptional function of E2F1. **A.** Luciferase experiments were performed in HeLa cells co-transfected with a plasmid encoding the firefly luciferase under the control of the wild type cyclin E promoter (WT E2F1) or under the control of the mutant cyclin E promoter in which the E2F1 response element had been mutated (mut E2F1); pRL-CMV encoding the Renilla luciferase for normalization and the indicated siRNAs. **B.** Luciferase experiments were performed in HeLa cells co-transfected with a plasmid encoding the firefly luciferase under the control of the SV40 viral promoter; pRL-CMV encoding the Renilla luciferase for normalization and the indicated siRNAs. **C.** Luciferase experiments were carried out in HeLa cells co-transfected with a plasmid encoding the firefly luciferase under the control of the SKP2 promoter; pRL-CMV encoding the Renilla luciferase for normalization and the indicated siRNAs. **D.** Luciferase experiments were carried out in stable HeLa cells expressing the indicated shRNAs (scrambled siRNA: shSCR or directed against Api5: shApi5) transfected with a plasmid encoding the firefly luciferase under the control of the wild type cyclin E promoter (WT E2F1) or under the control of the mutant cyclin E promoter in which the E2F1 response element has been mutated (mut E2F1); pRL-CMV encoding the Renilla luciferase for normalization and, when indicated, a plasmid (R-2HA2Flag-Api5) encoding Api5 whose RNA is resistant to the shApi5. **E.** Western blot analysis with the shSCR and shApi5 HeLa cell lines: detection of recombinant HA-Api5 resistant to shApi5 (HA), endogenous Api5 (Api5) and β-actin (Actin).

HeLa cells were transfected with a plasmid encoding the luciferase protein under the control of the wild type cyclin E promoter (WT E2F1) (Figure 3A) [37]. When the cells were co-transfected with an siRNA against E2F1, there was an 80% reduction in the luciferase activity (Figure 3A). A similar result was observed after Api5 knockdown. When the cells were co-transfected with both siRNAs at the same time, no further decrease in luciferase activity was observed (Figure 3A). To extend this experimentation, we mutated the E2F1 binding site in the cyclin E promoter (mut E2F1) and did identical experiments. For cells co-transfected with the mutated promoter (mut E2F1) and the scrambled siRNA, the luciferase activity was reduced by the same order of magnitude as for cells co-transfected with the WT E2F1 promoter and the specific E2F1 siRNA (Figure 3A) indicating that the E2F1 stimulation was abolished for the mut E2F1 construct. Interestingly, when the mutated promoter was co-transfected with the siRNA against Api5, there was no further decrease in the expression of the luciferase reporter. These data clearly show that the effect of Api5 depletion with the wild type cyclin E occurs through E2F1 transcription factor activity. Co-transfection of the mut E2F1 reporter plasmid with both siRNA (siE2F1 and siApi5) reinforced this conclusion since no additive effect was observed (Figure 3A). As a control, we used a reporter plasmid bearing luciferase downstream of the SV40 promoter. No significant modification of the luciferase acitivity was observed when the cells were cotransfected with E2F1, Api5 or both siRNAs (Figure 3B). These results revealed therefore that the Api5 effect could be specific for E2F1 regulated promoters. To support this conclusion we used another E2F1 responsive promoter, SKP2, that is regulated like the cyclin E promoter (Figure 3C) [37]. Co-transfection with a siRNA directed against E2F1, Api5 or both siRNAs potently reduced the activity of the SKP2 promoter reporter compared to the control condition (Figure 3C). Api5 depletion induced the same response and again, with both siRNAs co-transfections no cumulative effect was observed. This led us to conclude that Api5 positively controls E2F1 driven promoters most likely by directly or indirectly regulating E2F1 activity.

In order to minimize the variations and stress linked to multiple transfections, we generated stable HeLa cell lines expressing a scrambled shRNA or a specific Api5 shRNA, which considerably suppressed endogenous Api5 protein expression (Figure 3E, WB: Api5). Luciferase assays with reporters harboring a wild type (WT E2F1) or a mutated version (mut E2F1) of the cyclin E promoter were carried out in both stable cell lines (Figure 3D). Api5 overexpression was obtained by transfection of a plasmid encoding an HA tagged Api5 mRNA insensitive to the shApi5 (R-2HA2Flag-Api5) (Figure 3E WB: HA). As shown in Figure 3D, the cyclin E promoter activity (WT E2F1) was dramatically inhibited in the absence of Api5, thus recapitulating the observation shown in Figure 3A. Api5 overexpression in the shSCR cell line (R-2HA2Flag-Api5 expression) led to only a 1.5 fold increase in the cyclin E promoter activity. More interestingly, restoration of the expression of the HA-Api5 protein (R-2HA2flag-Api5) in the Api5 knockdown cell line (shApi5) fully restored cyclin E promoter activity. When the mutated cyclin E promoter was tested (mut E2F1) in control cell lines (shSCR) the same decrease in luciferase activity was obtained as previously observed as shown in Figure 3A. This decrease was not compensated by HA-Api5 ectopic expression, strengthening the view that Api5 acts on E2F1 responsive promoters through E2F1. Identical results were obtained for Api5 knockdown cell lines expressing or not HA-Api5.

To conclude, endogenous E2F1 fails to transactivate a cyclin E promoter in the absence of Api5 and this transactivation is dependent on the presence of an E2F1 binding site on the promoters (Figure 3C) demonstrating that the regulation of Api5/E2F1-dependent transcriptional activity is specific. Taken together, these observations clearly show that endogenous Api5 plays an essential positive role in E2F1 transcriptional activation, at least for G1/S phase transition genes.

### Api5 Modulates E2F1 Binding to Cell Cycle Related Promoters

**Figure 4:**
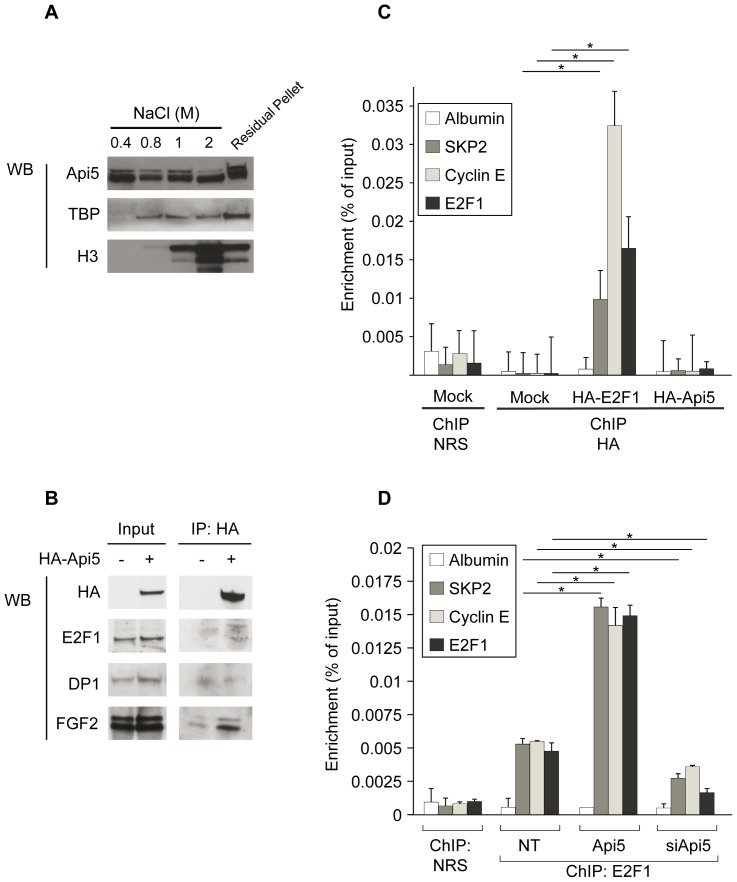
Api5 is a chromatin associated factor facilitating E2F1 association to specific target promoters. **A.** Saline chromatin extracts. Western blot analysis of chromatin extracts with Api5 antibody and TBP and histone H3 antibodies as control. Chromatin fractions were prepared by digesting nuclei with DNase and extracting with 0.4/0.8/1 and 2 M NaCl. Residual pellets correspond to proteins not extracted at 2 M NaCl. **B.** Api5 co-immunoprecipitation. HeLa cells were transfected with HA-tagged Api5 expression vector. Putative Api5 associated proteins were analyzed by Western Blot (WB) from co-immunoprecipitated protein complexes using anti-HA antibody. Western blot against high molecular weight forms of FGF2 was used as a positive control. **C.** HA-E2F1 and HA-Api5 ChIP experiments. HeLa cells were transfected with HA-tagged Api5 or HA-tagged E2F1 expression vectors (Mock = empty vector). The percentage of input reflects the enrichment of SKP2, cyclin E and E2F1 promoters after immunoprecipitation with an HA antiboby or with a normal rabbit serum (NRS). PCR amplified regions of the albumin promoter were used as a negative control. The ChIP results were obtained from 3 independent replicates; the error bars indicate the standard error. Statistical significance was determined by two-tailed Student’s *t* test (*, p<0.05). **D.** E2F1 ChIP experiment. Enrichment of SKP2, cyclin E and E2F1 promoters (% of input) after immunoprecipitation with E2F1 antiboby. Cells were transfected with an Api5 expression vector (Api5) or an siRNA directed against Api (siApi5). Chromatin immunoprecipitations were done using an anti-E2F1 polyclonal rabbit antibody (ChIP E2F1) or with normal rabbit serum (CHIP NRS). PCR amplified regions of the albumin promoter were used as a negative control. The ChIP results were obtained from 3 independent experiments; the error bars indicate the standard error. Statistical significance was determined by two-tailed Student’s *t* test (*, p<0.05).

Given the above results, we speculated whether Api5 could act directly or indirectly on E2F1 binding abilities by binding to E2F1 protein or in association with the chromatin within the E2F1 binding region. To answer this question, we first sought an association of Api5 with chromatin by performing sodium chloride extraction experiments on HeLa cell nuclei (Figure 4A). Api5 appeared to be a chromatin-associated nuclear factor *in vivo* as it biochemically behaves like other chromatin associated factors such as TBP (Tata Binding Protein) or histone H3 even if it starts to eluate at a lower sodium chloride concentration (0.4 M). The fact that Api5 is a chromatin associated factor behaving biochemically like a transcription factor, led us to consider the possibility that it could be associated with E2F1 in a protein complex.

To go further into the study of the functional relationship between Api5 and E2F1 we investigated a potential interaction between Api5 and E2F1 by co-immunoprecipitation assays. HeLa cells were transfected or not with a plasmid encoding an HA tagged Api5 (HA-Api5). After immunoprecipitation using an HA-antibody, the co-immunoprecipitated products were analyzed by Western blot (Figure 4B). Fibroblast growth factor 2 was recovered as previously described [25], but neither E2F1 nor its binding partner DP1 could be detected. This indicates that there is unlikely to be any physical interaction between Api5 and E2F1 and that they are not part of a complex with DP1 in HeLa cells.

We next explored the possibility that Api5, even if not interacting physically with E2F1, could be associated on the E2F1 target gene promoters. For this, we transfected HeLa cells with a plasmid encoding HA-E2F1 or HA-Api5 and performed a chromatin immunoprecipitation (ChIP) with an anti-HA antibody (Figure 4C). Our ChIP data indicated a clear binding of HA-E2F1 on the target promoters SKP2, E2F1 and cyclin E but not on the albumin promoter used as a negative control. These results are in accordance with the literature [32] [36] [38]. However, we failed to detect any Api5 binding on any E2F1 regulated promoters (SKP2, E2F1 or cyclin E). Similar results were obtained with ChIP experiments against the endogenous Api5 and E2F1 proteins (data not shown).

Finally we tested the possibility that E2F1 recruitment on its target promoters could be affected by Api5. We carried out ChIP experiments against E2F1 on the same promoters in the context where Api5 was overexpressed (Api5) or knocked-down by siRNA (siApi5) (Figure 4D). Relative to non-transfected cells, Api5 overexpression induced an increased recruitment (∼3 fold) of the endogenous E2F1 transcription factor on the three E2F1 responsive promoters SKP2, cyclin E and E2F1. In agreement, Api5 knock-down induced a decrease in E2F1 binding (∼2 fold) to these three promoters. As expected, no E2F1 binding to the albumin promoter was observed.

These results clearly demonstrate that Api5 contributes positively and indirectly to the recruitment of E2F1 on its target promoters. Collectively, our findings provide evidence of a functional relation between Api5 and the E2F1 transcriptional activity E2F1 target genes during the G1/S phase.

## Discussion

In the present study we extend the characterization of Api5 protein function and its existing relationship with the E2F1 transcription factor during the cell cycle. We show that Api5 expression, like E2F1, is periodically regulated during the cell cycle. However, no reciprocal regulation between Api5 and E2F1 was observed for their respective expression. Interestingly, Api5 siRNA is able to mimic the effect of an E2F1 siRNA on E2F1 target genes involved in the G1/S phase transition by down-regulating their expression. This result has functional consequences: Api5 siRNA treatment is able to block the G1/S phase transition of the cell cycle, leading to a delay in cell growth, similar to E2F1 interference. Moreover, the lack of an additive effect of the double Api5/E2F1 knock-down on cell cycle proliferation, expression and reporter gene experiments, (Figures 1C and 1D, 2F and 4) suggests that these two factors belong to the same regulatory pathway. Altogether, Api5 participates in a positive regulation at the transcriptional level of the E2F1 target genes involved during the G1/S phase transition. This effect is indirect and affects E2F1 recruitment to its target as demonstrated by chromatin immunoprecipitation experiments.

At the molecular level, chromatin immunoprecipitation experiments showed that Api5 acts by promoting the recruitment of E2F1 onto specific target promoters. This effect is most likely indirect, as Api5 and E2F1 do not belong to the same protein complex as demonstrated by the co-immunoprecipitation experiments. As we show that Api5 is a chromatin associated factor, one possibility was that Api5 could localize to the E2F1-target promoters, and participate to their transcriptional regulation without any physical contact with E2F1. This hypothesis has been tested by doing a chromatin immunoprecipitation assay using an anti-Api5 antibody. Unfortunately, no binding of Api5 was detected in the vicinity of the E2F1 binding sites. Several sets of primers were used in this chromatin immunoprecipitation assay, but failed to detect any Api5 binding up to 5 kb upstream and 2 kb downstream of the transcription start site of the Cyclin E gene (data not shown). These results are in accordance with our data showing that E2F1 and Api5 did not co-localize in immunofluorescent staining experiments (Figure 1B, merge).

Another possibility to explain the indirect effect of Api5 on E2F1 binding to specific target genes could be the modulation of E2F1 post-translational modifications that could modify E2F1 binding properties to DNA. Among them, induction of phosphorylation by Cdk4/6-Cyclin D complexes at serine residues 332 and 337, which stabilizes E2F1 and prevents its binding to pRb, should be considered as well as the phosphorylation by the Cdk2/Cyclin A complex at serine 375, which reduces E2F1 DNA binding. An induction of such post-translational modification on E2F1 cannot account for the increased binding capacity of E2F1 that we observed when Api5 was over-expressed. However, the reverse hypothesis could also be considered: i.e., an inhibition of serine 375 phosphorylation that could probably lead to an increase in E2F1 binding to its target genes. Finally, acetylation of lysine residues 117, 120, and 125 by the complex CBP/P/CAF can strongly enhance DNA binding to its target genes and stabilize E2F1 further [39]. However, acetylation of E2F1 only occurs in conditions of DNA damage where it has been shown that acetylated E2F1 is recruited to specific target genes like p73 to induce apoptosis [40]. Nevertheless, this mechanism could not explain our observations since it is clearly demonstrated that E2F1 acetylation is related to its apoptotic effects [40] whereas Api5-(over)expression generates the opposite effect and protects cells against apoptosis ([24] and our unpublished results).

Furthermore, a study by Ahel and collaborators, [41] showed that Api5 interacts with the chromatin remodeling enzyme ALC1 (Amplified in Liver Cancer 1) which is a member of the SNF2 (Sucrose Non Fermenting 2) family. Interestingly, it has been previously reported that ALC1 is able to promote G1/S phase transition in hepatocarcinoma cells [42]. Thus, it could be of interest to consider the possibility that ALC1, or a protein associated to the ALC1 complex could be the link between Api5 and the ability of E2F1 to reach and activate specific target promoters. Hence, epigenetic modifications in the E2F1 target promoters induced by Api5 could regulate the access of E2F1 to the chromatin, and thus explain the modulation of E2F1 binding to these promoters that we observed.

Another prospect is that Api5 could activate the expression of a gene whose product could enhance the binding and activation of E2F1 to specific targets during G1/S phase transition. To our knowledge, this kind of protein has not yet been identified. This indirect pathway could be considered since it has been postulated that Api5 has trans-activation capacities [25]. In addition, we did immunocytochemistry experiments that strongly suggest that Api5 associates with euchromatin as revealed by its distribution at the center of the cell nuclei (Figure S3). Furthermore, sodium chloride extraction experiments indicate that Api5 is a chromatin-associated nuclear factor *in vivo*. Unfortunately, no Api5 target gene has yet been identified and such characterization will be of considerable interest to explain the key role of Api5 in E2F1 dependent control of G1/S transition.

## Material and Methods

### Cell Lines, and Transfections

HeLa (ATCC number CCL-2) and H1299 (ATCC number CRL-5803) cell lines were grown in DMEM media (Lonza) supplemented with 10% fetal bovine serum (FBS), 1% glutamine (Gibco) and antibiotics, at 37°C in a 5% CO_2_ humidified atmosphere. Cell synchronization by double-thymidine (Sigma) block was performed as previously described [43]. Cells were starved (in 0% FBS) for 36–48 h, then allowed to re-enter the cell cycle by addition of 10% FBS growth medium.

Cells were transfected using JetPEI for DNA constructs HA-Api5, 2HA2FlagApi5 and HA-E2F1 and InterferIN (for siRNAs) transfection reagents (Polyplus transfection) according to the manufacturer’s instructions. Api5 and E2F1 plasmid constructs upon request. siRNAs targeting sequences were as follow : *FIF* 5′-GGCCAGCATAAAGATGCCTAT-3′; *E2F1* 5′-GUCACGCUAUGAGACCUCATT-3′.

Stable HeLa cell lines were generated by infecting the cells with lentiviral particles produced in HEK293FT (Invitrogen #R700-07) with the two helper plasmids pLvVSVg and pLvPack (Sigma Aldrich) plus a lentiviral plasmid. shRNA against Api5 originates from a lentiviral plasmid MISSION® pLKO.1-puro (clone : NM_006595.2-278s1c1) containing the sequence CCGGGCAGCTCAATTTATTCCGAAACTCGAGTTTCGGAATAAATTGAGCTGCTTTTTG and shSCR originates from a lentiviral plasmid MISSION® pLKO.1-puro Non-Target shRNA Control Plasmid DNA (ref:SHC016-1EA) containing the sequence CCGGCAACAAGATGAAGAGCACCAACTCGAGTTGGTGCTCTTCATCTTGTTGTTTTTG, both from the Sigma Aldrich Company.

### Cell Cycle Analysis by FACS

Cells were collected, washed with PBS and fixed overnight at 4°C in 70% Ethanol, diluted in PBS. The next day, cells were washed with PBS and incubated for 30 min in PBS with 0,1% Triton X-100, RNaseA (0,2 mg/ml) and propidium iodide (20 µg/ml), at room temperature. The protocol was adapted from Darzynkiewicz *et al*., in Current Protocols in Cell Biology [44]. Stained cells were analyzed on a LSRII flow cytometer (BD Biosciences) with exclusions of doublets. Analysis of the results was performed with FlowJo software.

### Western Blot Analysis

Cells were collected after 72 hours of siRNA transfection or 48 hours of expression vector transfection,resuspended in Triton X-100 sample buffer and sonicated. 30 µg of proteins were resolved in 4–20% denaturing polyacrylamide gels (Thermo Scientific) and transferred onto a nitrocellulose membrane (Amersham). Immunoblotting were performed using anti-API5 antibody (ab56392, Abcam), E2F1 (C-20): sc-193 antibody (Santa Cruz), anti-OPA1 612606 antiboby (BD Transduction Laboratories), cyclin A (H-432): sc-751 antibody (Santa Cruz), histone H3 (ab1791) Abcam, Rb sc-102 (Santa Cruz), monoclonal anti-HA antibody H9658 (Sigma) and monoclonal anti-β-actin A5441 antibody (Sigma). The signal was detected using enhanced chemiluminescence detection reagent (Amersham). Densitometric analyses were performed with Image J.

### RNA Extraction and Quantification Using Real-time PCR

Total RNA was extracted using the TriZol reagent protocol (Invitrogen). Reverse transcription was performed with 2 µg of total RNA using RevertAid H Minus First Strand cDNA Synthesis Kit (Fermentas) and oligo(dT) primers. For quantification of mRNAs, real-time PCR was performed using SsoFast EvaGreen Supermix (Bio-Rad) with HPRT1 and GUSB mRNAs as endogenous control references (for oligonucleotide sequences see Table S1). Assays were performed on 7500 Fast Real-Time PCR System (Applied Biosystems). All the real-time PCR analyses were done in triplicate.

### Immunofluorescence Microscopy

Coverslips were washed in PBS and cells fixed in 3% Paraformaldehyde for 7 minutes and finally washed two times 5 minutes in PBS with 50 mM of NH_4_Cl. Cells were permeabilized with 0,5% Saponin for 10 minutes at 37°C. Blocking was carried out by washing two times 5 minutes in blocking buffer (PBS, 0,5% Saponin and 3% BSA). Primary and secondary antibody incubation were carried out at room temperature for 1 hour in blocking buffer and washes were performed using blocking buffer. Primary antibodies used were: mouse monoclonal anti-API5 (Abnova) and rabbit polyclonal anti-E2F1 (sc-193, Santa Cruz). Secondary antibodies used were Alexa Fluor 647 donkey anti-mouse IgG and Alexa Fluor 488 goat anti-rabbit IgG (Molecular Probes). Cells were counterstained with propidium iodide (PI) before mounting. Images were obtained using LSM510 Confocal Laser Scanning microscope equipped with an Axiovert 200M inverted microscope (Carl Zeiss) and a 40X objective lens (C-Apochromat, 1,2 W, Oil), using three laser lines (488, 543 and 633 nm). Subsequent images handling was carried out in Adobe Photoshop CS.

### Luciferase Reporter Assay

HeLa cells were co-transfected with the indicated promoter constructs pGL2-cyclin E (WT or mut E2F1) (300 ng), pGL2-SKP2 (300 ng) or pGL2-SV40 (50 ng) and pRL-CMV (5 ng) using JetPEI reagent according to the manufacturer protocol (Polyplus transfection) and, when indicated with the corresponding siRNAs with InterferIN transfection reagent (Polyplus transfection). Twenty-four hours after transfection, cells were lyzed in Passive Lysis Buffer (PLB) and firefly luciferase activity was measured in a LB960 luminometer (Berthold) according to manufacturer’s recommendations and by using the dual reporter assay kit (E1960) (Promega).

### Immunoprecipitation and Western Blot Analysis

For preparation of nuclear extracts, 5.10^7^ control HeLa cells or cells transfected with HA-FIF (with Hemagglutinin tag) expression vector were washed in PBS and resuspended in 4 ml of chromatin fractionation buffer (0,15 M NaCl;10 mM MgCl2; 10 mM CaCl2; 15 mM Tris, pH 7,5; 0,1% Tween 20; protease inhibitors). Cells were ruptured by using Ultra-Turrax (IKA) in the presence of 0,1% of NP-10. Nuclei were collected by centrifugation, resuspended in Lysis Buffer (150 mM NaCl, 1% 100X Triton, 50 mM Tris HCl pH = 8) and sonicated. Co-Immunoprecipitation was performed using mMACS HA Tagged Protein Isolation Kit (Miltenyi Biotec). Western Blot analysis was achieved as previously described, using E2F1 (C-20): sc-193 antibody (Santa Cruz), DP1 (SPM178) antibody (GeneTex), FGF2 (147): sc-79 (Santa Cruz) and monoclonal anti-HA antibody H9658 (Sigma).

### Preparation of Chromatin Fractions

3 × 10^7^ HeLa cells were washed in PBS and resuspended in 1 ml of chromatin fractionation buffer (0,15 M NaCl; 10 mM MgCl2; 10 mM CaCl2; 15 mM Tris, pH 7,5; 0,1% Tween 20; protease inhibitors). Cells were ruptured by using Ultra-Turrax Ultra-Turrax (IKA) in the presence of 0.1% of NP-10. After centrifugation (1500 rpm, 10 min at 4°C), nuclei were digested with DNase 1 (0.2 µg/µl) for 10 min at 30°C and pelleted by a brief centrifugation. Chromatin fractions were prepared by adding NaCl, to a final concentration of 0.4 M, to the nuclear pellets resuspended in chromatin fractionation buffer. After 30 min at 4°C, the nuclei were centrifuged at 13000 rpm for 10 min, and the supernatant (chromatin fraction 0,4 M) was saved. Chromatin fractions 0.8/1/2 M were similarly prepared by adding NaCl to the desired final concentration. The final pellet was saved as residual pellet. Mouse monoclonal [1TBP18] to TBP (ab818, Abcam) and rabbit polyclonal to Histone H3 (ab1791, Abcam) antibodies were used to validate the chromatin fractions by Western Blot analysis.

### Chromatin Immunoprecipitation Assay

Chromatin Immunoprecipitation assay was performed using Transcription Factor ChIP Kit (diagenode), following manufacturer’s protocol. The immunoprecipitation of DNA-protein complexes was achieved with antibodies directed against E2F1 (sc-193, Santa Cruz) or HA-tag (ab9110-100, Abcam). We measured the abundance of the candidate sequences by quantitative PCR amplification, as previously described (for oligonucleotide sequences see Table S1). We performed immunoprecipitations with normal rabbit serum, PCR amplified regions of the albumin promoter and the 1,5 kb region upstream the interested region as negative controls (data not shown). Values reflecting chromatin enrichment are reported as the percent of input.

## Supporting Information

Figure S1 **H1299 cells were enriched at G1 phase by a double thymidine block, washed and released through the cell cycle.** Every hour, cells were collected and DNA content was analyzed after propidium iodide (PI) staining, with a LSRII flow cytometer. Cell number (counts) was plotted against DNA content (PI fluorescence).
(PDF)

Figure S2 **H1299 cells were transfected with Api5 or E2F1 siRNA, or with both siRNAs.** After propidium iodide (PI) DNA staining, cell cycle distribution analysis was carried out with a LSRII flow cytometer. Cell number (counts) was plotted against DNA content (PI fluorescence).
(PDF)

Figure S3 **Api5 associates with euchromatin in the nucleus. A.** Colocalization of Api5 with euchromatin was observed by immunostaining of HeLa cells. Endogenous Api5 (green) is mainly localized into euchromatin as it is excluded from nucleoli and from the periphery of the nuclei (heterochomatin). [Scale bar 20 µm]
(PDF)

Table S1 **Oligonucleotide sequences.** (5′ to 3′)
(PDF)
