# Supplementary figures and images for: Api5 Contributes to E2F1 Control of the G1/S Cell Cycle Phase Transition
Source: PLoS One. 2013 Aug 7;8(8):e71443. doi: 10.1371/journal.pone.0071443 (PMC3737092; doi:10.1371/journal.pone.0071443)

Figure S1

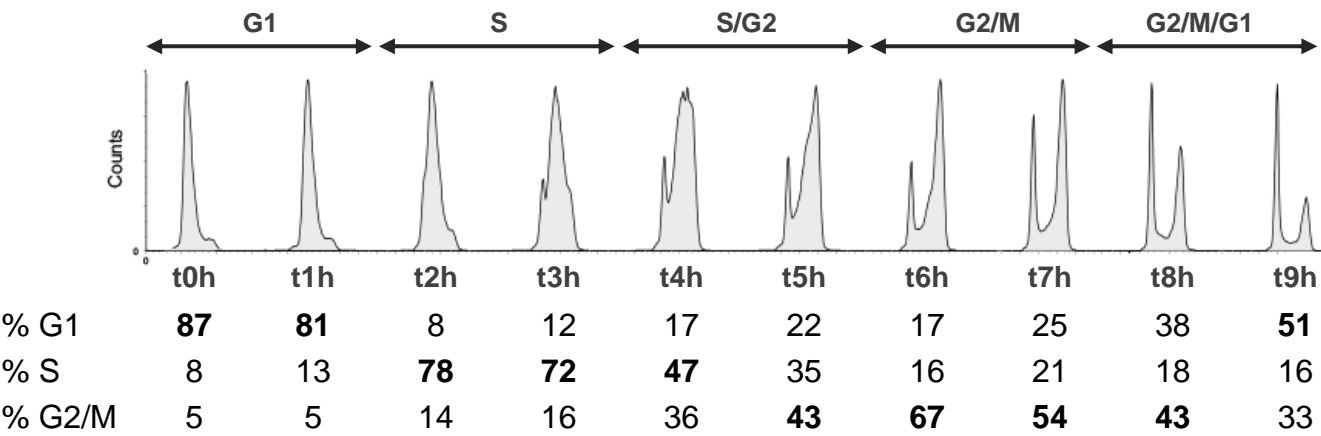

Supplement: Figure S1 — H1299 cells were enriched at G1 phase by a double thymidine block, washed and released through the cell cycle. Every hour, cells were collected and DNA content was analyzed after propidium iodide (PI) staining, with a LSRII flow cytometer. Cell number (counts) was plotted against DNA content (PI fluorescence). (PDF) [file pone.0071443.s001.pdf]

**Figure S2**

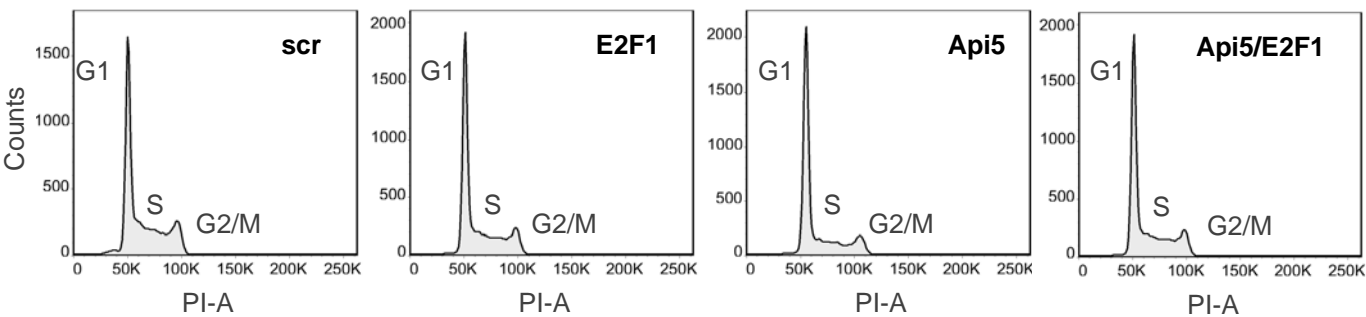

Supplement: Figure S2 — H1299 cells were transfected with Api5 or E2F1 siRNA, or with both siRNAs. After propidium iodide (PI) DNA staining, cell cycle distribution analysis was carried out with a LSRII flow cytometer. Cell number (counts) was plotted against DNA content (PI fluorescence). (PDF) [file pone.0071443.s002.pdf]

Figure S3

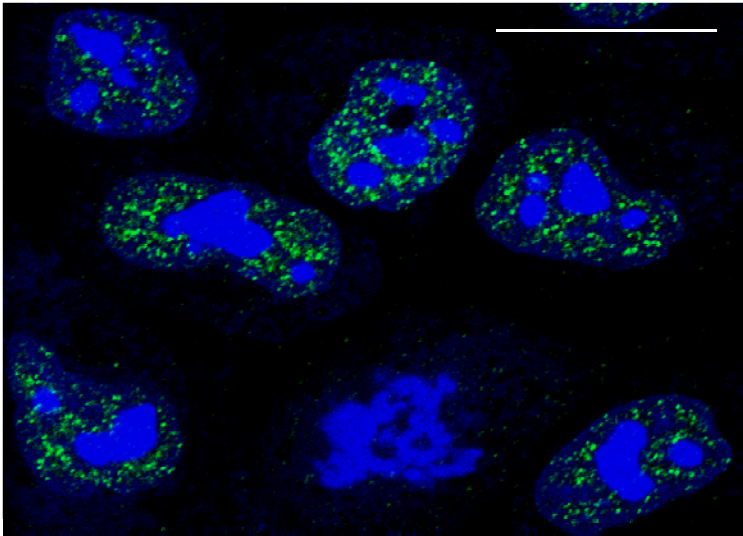

Supplement: Figure S3 — Api5 associates with euchromatin in the nucleus. A. Colocalization of Api5 with euchromatin was observed by immunostaining of HeLa cells. Endogenous Api5 (green) is mainly localized into euchromatin as it is excluded from nucleoli and from the periphery of the nuclei (heterochomatin). [Scale bar 20 µm] (PDF) [file pone.0071443.s003.pdf]
