# Supplementary material for: Api5 Contributes to E2F1 Control of the G1/S Cell Cycle Phase Transition
Source: PLoS One. 2013 Aug 7;8(8):e71443. doi: 10.1371/journal.pone.0071443 (PMC3737092; doi:10.1371/journal.pone.0071443)
Supplement: Table S1 — Oligonucleotide sequences. (5′ to 3′) (PDF) [file pone.0071443.s004.pdf]

Table S1. Oligonucleotide sequences. (5' to 3')

**Primers for cDNA amplification**	**Forward**	**Reverse**
Api5	CCGACAGTAGAGGAGCTTTACCGCA	AGGCATCTTTATGCTGGCCCACT
E2F1	CGACCTGAACTGGGCTGCCG	CGCCCACTGTGGTGTGGCTG
Cyclin E	AAGCACTTCAGGGGCGTCGC	GGTGAGGAGCCCACTGGGGA
Cyclin A	AGGGCTGCTAACTGCAAATGGGC	CTCTTGGACCCCACAGTCAGGGA
Cyclin D1	GGGTGCTGCGGGCCAT	TCGCACTTCTGTTCCTCGCA
P16 INK4a	AGTTACGGTCGGAGGCCGAT	ACGGGTCGGGTGAGAGTGG
CDK2	CAGCTGTGGACATCTGGAGCCTG	TCCTGGCCACACCACCTCATCTG
Cdc25A	GAACTCTGCCCCAGCTCGGAT	GGTCCACGAAGCCATCATCCTCA
GUS B	GATGACATCACCGTCACCACCAGC	CCCAGTCCCATTCGCCACGACT
HPRT1	GGCTCCGTTATGGCGACCCG	AAACACCCTTTCCAAATCCTCAGCA
**Primers for gDNA amplification**	**Forward**	**Reverse**
Albumin	TACATTGACAAGGTCTTGTGGAG	TGGGGTTGACAGAAGAGAAAAGC
SKP2	CGGGACGGAAACTACAATTC	AAGCCTAGCAACGTTCCATC
Cyclin E	GCCGCCCGCCGTGTTTACAT	GGCGCTGGAGCGGCAAAAAG
E2F1	AGGAACCGCCGCCGTTGTTCCCG	CTGCCTGCAAAGTCCCGGCCACTT
SKP2 +1,5Kb	TCCCTCCTTAATAGCTCCCCATCCCC	CTGGGAGGCACTCGTTCGCC
Cyclin E +1,5Kb	TTGCAGAGCAGCAGCCAGGG	CGGCGCACTGCGTTGAAACC
E2F1 +1,5Kb	AGGGCTTTTAGGGGCCCTCCC	CACCTGGCCCACCCCTGGTA
**Primers for mutagenesis** of cyclin E	**Forward**	**Reverse**

promoter		
mutE2F1	CAGCCCTCGGGGCGGGGAATTCGGGCGGGACGGGGCCG	CGGCCCCGTCCCGCCCGAATTCCCCGCCCCGAGGGCTG
pGL	GL1 : TGTATCTTATGGTACTGTAACTG	GL2: TGTATCTTATGGTACTGTAACTG
**Primers for mutagenesis** of Api5 cDNA	**Forward**	**Reverse**
Resistance to shApi5	GAAAGGTGGTACTAAGGAAAAGCGATTAgcCgcAca GttCatCccTaaGtTCTTTAAaCATTTTCCAG	CTGGAAAATGtTTAAAGAaCttAggGatGaaCtgTgc GgcTAATCGCTTTTCCTTAGTACCACCTTTC
